# Assembly and Annotation of Red Spruce (*Picea rubens*) Chloroplast Genome, Identification of Simple Sequence Repeats, and Phylogenetic Analysis in *Picea*

**DOI:** 10.3390/ijms232315243

**Published:** 2022-12-03

**Authors:** Rajni Parmar, Federica Cattonaro, Carrie Phillips, Serguei Vassiliev, Michele Morgante, Om P. Rajora

**Affiliations:** 1 Faculty of Forestry and Environmental Management, University of New Brunswick, 28 Dineen Drive, Fredericton, NB E3B 5A3, Canada; 2 IGA Technology Services, Via Jacopo Linussio, 51, 33100 Udine, Italy; 3 Forest Genetics and Biotechnology Group, Department of Biology, Dalhousie University, Halifax, NS B3H 4J1, Canada; 4 ACENET, University of New Brunswick, Fredericton, NB E3B 5A3, Canada; 5 Laboratory of Plant Genomics, University of Udine, 33100 Udine, Italy

**Keywords:** organellar genome, conifers, Pinaceae, plastid genome, genome sequences, genome assembly and annotation, microsatellites, comparative genome analysis, phylogeny

## Abstract

We have sequenced the chloroplast genome of red spruce (*Picea rubens*) for the first time using the single-end, short-reads (44 bp) Illumina sequences, assembled and functionally annotated it, and identified simple sequence repeats (SSRs). The contigs were assembled using SOAPdenovo2 following the retrieval of chloroplast genome sequences using the black spruce (*Picea mariana*) chloroplast genome as the reference. The assembled genome length was 122,115 bp (gaps included). Comparatively, the *P. rubens* chloroplast genome reported here may be considered a near-complete draft. Global genome alignment and phylogenetic analysis based on the whole chloroplast genome sequences of *Picea rubens* and 10 other *Picea* species revealed high sequence synteny and conservation among 11 *Picea* species and phylogenetic relationships consistent with their known classical interrelationships and published molecular phylogeny. The *P. rubens* chloroplast genome sequence showed the highest similarity with that of *P. mariana* and the lowest with that of *P. sitchensis*. We have annotated 107 genes including 69 protein-coding genes, 28 tRNAs, 4 rRNAs, few pseudogenes, identified 42 SSRs, and successfully designed primers for 26 SSRs. Mononucleotide A/T repeats were the most common followed by dinucleotide AT repeats. A similar pattern of microsatellite repeats occurrence was found in the chloroplast genomes of 11 *Picea* species.

## 1. Introduction

Chloroplast is a characteristic and an essential plant cell organelle in higher plants and algae because chloroplasts are the sites of photosynthesis, which is a life-sustaining process on the planet earth. Plant chloroplasts have their own genomes, which are predominantly uniparentally inherited, maternally in angiosperms, for example in *Populus* [1], and paternally in conifers, for example in *Pinus* [2]. Their genes are involved in photosynthesis in conjunction with the nuclear genes. In comparison to mitochondrial and nuclear plant genomes, chloroplast (cp) genomes have a slower evolutionary rate, and thus have more conserved gene number, gene content, composition, and organization. These features make the plant chloroplast genomes and their genes and sequences an excellent source of genetic markers for phylogenetics, phylogenomics, systematics, phylogeography, biogeography, population and evolutionary genetics, and pollen and seed dispersal studies and applications. Indeed, chloroplast DNA markers and genes have been widely used for such studies over the past several decades [3]. Therefore, understanding the structure, gene content, and sequences of the plant chloroplast genomes is of high basic and applied importance.

Chloroplasts were derived from ancient single photosynthetic cyanobacterium engulfed by eukaryotic cells [4,5,6,7]. Subsequently, the genome of the endosymbiont shrank after host-endosymbiont coevolution for years [8]. A few genes were lost, and a few were transferred to the host nuclear genome. In the present chloroplast proteome, ~3000 proteins exist, the majority of which are encoded by the nuclear genome and post-translationally transported to chloroplasts [9]. Besides photosynthesis processes, several metabolites, such as, amino acids, nucleotides, fatty acids, phytohormones, and vitamins are also synthesized via various biochemical processes in the chloroplast. Many of these metabolites are important for maintaining communication during biotic and abiotic stress conditions between different parts of the plants [10,11]. Therefore, chloroplast genome analysis also helps to understand the interaction between the nuclear and chloroplast genomes [9].

Spruce (*Picea* Mill.) species are the major components of the boreal, temperate, montane, and subalpine forests throughout the Northern Hemisphere. For example, transcontinental black spruce (*Picea mariana* (Mill.) B.S.P.) and white spruce (*Picea glauca* (Moench) Voss) are predominant species of the Canadian boreal forest [12]. In their range, spruce species are highly ecologically, economically, and environmentally important, serving as huge carbon sinks. In North America, *P. mariana*, *P. glauca*, red spruce (*Picea rubens* Sarg.), Sitka spruce (*Picea sitchensis* (Bong.) Carr.) and Englemann spruce (*Picea engelmannii* Parry ex Engelm.) are economically and ecologically the most important species [12,13], whereas, in Europe, Norway spruce (*Picea abies* L.) is ecologically important and has high economic importance for timber and pulp and paper industries [14].

The majority of the *Picea* species are morphologically similar with incomplete sorting of the lineage and complex phylogeny due to interspecific introgression resulting in difficulty in unambiguous identification of the species [15,16,17]. The use of the chloroplast genome sequences could help in resolving the phylogeny and systematics of the genus *Picea*, as well as assist in understanding phylogeography, pollen gene dispersal, and organellar genomic diversity in *Picea*. Indeed, chloroplast DNA markers and genes have been used for examining phylogeny and phylogeography in the genus *Picea* [15,16,17]. Until now, the complete chloroplast genomes of a few *Picea* species are available, including more recent chloroplast genomes of North American *P. mariana* (123,961 bp) [18], *P. glauca* (123,421 bp) [19], *P. sitchensis* (124,049 bp) [20], *P. engelmannii* (123,542 bp) [21], and European *P. abies* (124,084 bp) [22]. The chloroplast genome sequence and annotation of *P. rubens* have not yet been reported.

*Picea rubens* is an important late-successional, shade-tolerant species of temperate forests of eastern Canada and the northeastern United States [23]. It has relatively low genetic diversity [24] and a narrow ecological niche that makes it sensitive to climate and environmental changes [25]. *P. rubens* has declined in the southern parts of its range, which has been associated with industrial air pollution [26,27]. In the northern range, introgressive hybridization occurs with sympatric *P. mariana* [28,29,30]. However, the extent of hybridization and evolutionary relationships between *P. rubens* and *P. mariana* are not very clear. The comprehensive chloroplast genomic resource and SSR markers are valuable for future evolutionary studies and can facilitate the resolution of these relationships between *P. mariana* and *P. rubens*.

In the present study, we have sequenced, assembled, and annotated the chloroplast genome of *P. rubens* by extracting DNA from isolated chloroplasts and sequencing the chloroplast genome using single-end short (44 bp) Illumina sequences. We have identified microsatellites (Simple Sequence Repeats, SSRs) in the assembled genome and designed flanking primers. We have also performed a comparative genome analysis to examine the sequence synteny, genome divergence, and pattern of microsatellite repeats occurrence in the chloroplast genomes of 11 *Picea* species: *Picea sitchensis*, *P. engelmannii*, *P. glauca*, *P. chihuahuana*, *P. neoveitchii*, *P. abies*, *P. asperata*, *P. crassifolia*, *P. jezoensis*, *P. mariana*, and *P. rubens*. Furthermore, we have examined phylogenetic relationships among these 11 *Picea* species based on their whole chloroplast genome sequences.

## 2. Results and Discussion

### 2.1. Chloroplast Genome Features and Gene Content

Illumina sequencing provided a total of 2,577,052 high-quality reads and an estimated genome coverage of 928.55×. An assembly of *Picea rubens* sequences using SOAPdenovo2 resulted in a total of 2505 contigs with the longest contig of 17,176 bp. The final scaffolding of the chloroplast-specific sequences in the assembly obtained after the alignment of the assembled contigs to the reference *P. mariana* chloroplast genome and gap filling resulted in the *Picea rubens* draft chloroplast genome of 122,115 bp with a few gaps (Ns), 0 misassemblies and 38.96% GC content. Quality parameters of the *P. rubens* chloroplast genome, estimated after mapping the assembled scaffolds using QUAST with the *P. mariana*, *P. glauca*, *P. sitchensis*, *P. abies*, *P. engelmannii*, *P. chihuahuana*, *P. neoveitchii*, *P. asperata*, *P. crassifolia*, and *P. jezoensis* chloroplast genomes [20,31], are presented in Table 1. The GC content of the protein-coding regions of the *P. rubens* chloroplast genome was found to be almost the same as reported for other members of the *Picea* genus: *P. sitchensis* (38.7%), *P. engelmannii* (38.74%), *P. glauca* (38.74%), *P. chihuahuana* (38.7%), *P. neoveitchii* (38.77%), *P. abies* (38.72%), *P. asperata* (38.71%), *P. crassifolia* (38.71%), *P. jezoensis* (38.8%), and *P. mariana* (38.7%) (Supplementary Table S1) as well as in other members of the Pinaceae family [32,33]. After QUAST analysis, InDels in the *P. rubens* chloroplast genome in comparison to the chloroplast genomes of the 10 above *Picea* species were found to be the lowest with the *P. mariana* and the highest with the *P. sitchensis* chloroplast genomes (Table 1). Likewise, InDels of <=5 bp were also found the lowest in comparison with *P. mariana* and the highest in comparison with *P. sitchensis* (Table 1). Overall, the InDels data reveals that *P. rubens* is more closely related to *P. mariana* and *P. abies* than to *P. glauca* and *P. sitchensis*. This is consistent with known close relationships between *P. rubens* and *P. mariana* [28,29,30,34,35].

Because after the assembly with SOAPdenovo2, the assembled contigs were mapped to the *P. mariana* reference genome and chloroplast genome-specific sequences were extracted, we believe that only chloroplast genome-derived sequences have been co-assembled into a single scaffold, resulting in the 122,115 bp draft [including gaps (Ns)] chloroplast genome assembly of *P. rubens*. This length of the *P. rubens* chloroplast genome is shorter than that reported for sympatric *P. mariana* (123,961 bp) and *P. glauca* (123,421) as well as allopatric *P. sitchensis* (124,049) and *P. abies* (124,084). From the published information, it is apparent that the chloroplast genomes of the *Picea* species are about 123 to 124 kb in size. Assuming that the size of the chloroplast genome of *P. rubens* is in this range, we could state that the *P. rubens* chloroplast genome reported in our study is likely not complete and could be considered as a draft or near-complete genome. The shorter assembled chloroplast genome of *P. rubens* is likely due to the presence of gaps in the final assembly resulting from the use of single-end short sequence reads (1 × 44 bp) for scaffolding. From mapping the assembled contigs of *P. rubens* to the chloroplast genome of *P. mariana*, the missing regions in the chloroplast genome assembly of *P. rubens* could be identified (Supplementary Figure S1; Supplementary Material File S2). The results show that the short missing regions in the assembly, represented by Ns, are not located in a particular region of the *P. rubens* chloroplast genome but are distributed over the entire genome. This indicates that the gaps are likely due to very short single-end sequences used for assembly. Nevertheless, the genome assembly reported here provides a good foundation for completing and polishing the *P. rubens* chloroplast genome using longer and/or pair-end sequencing reads.

**Figure 1:**
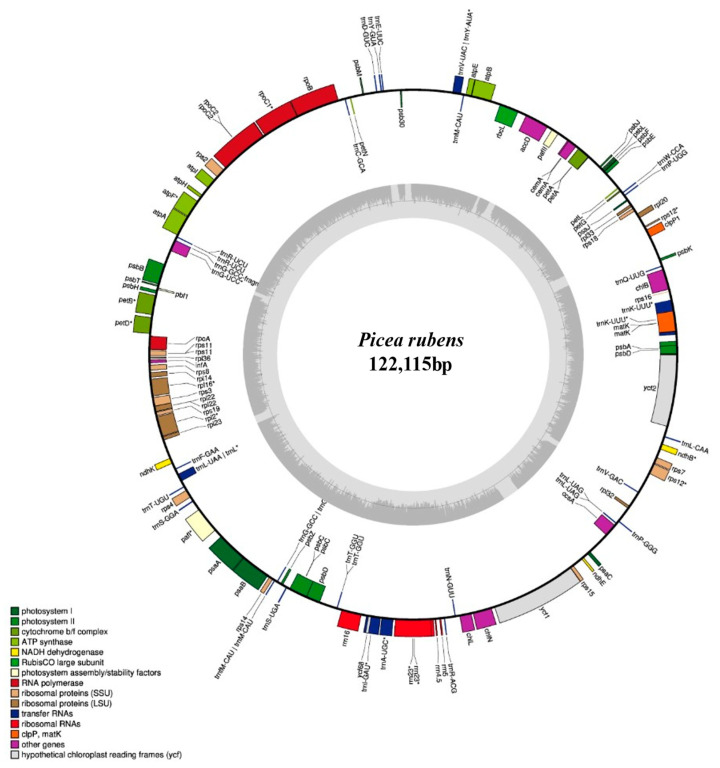
Near-complete chloroplast genome of *Picea rubens*, annotated using GeSeq v1.79 and organized using OGDRAW version 1.3.1 [(*) asterisk represents intron-containing genes in organelle genomes]. The *ndh* genes in the figure are truncated pseudogenes.

We were able to annotate 107 genes using GeSeq v1.79 with the chloroplast genomes of 11 *Picea* species as the reference (Table 2), including 69 protein-coding genes, 28 tRNAs, 4 rRNAs, and a few pseudogenes (Figure 1 and Table 2). Among the photosystem II genes, the *psbD* gene was identified. Further, among the small ribosomal subunits, fragmented copies of *rps12* (trans-splicing) and *rps16* [pseudogene, missing exon(s), and no start codon] were identified. The presence of the non-functional *rps16* gene fragments, which is similar to the previous findings in *Pinus thunbergii*, *Picea crassifolia*, and *Picea asperata*, further supports the fact of common loss of this gene in Pinaceae [36,37]. One tRNA gene, *trnG-GCC*, was found annotated at the same position in the *P. rubens* and *P. mariana* chloroplast genomes, which further supports the chloroplast genome similarity between these two species. The *ndhB* (pseudogene, with missing exons), *ndhE* (pseudogene, no start codon possible pseudogene, premature stop codon), and *ndhK* (truncated pseudogene) were also identified in our present assembly of the *P. rubens* chloroplast genome. Similar results have also been reported in *Pinus* of Pinaceae and *Welwitschia* of Gnetophytes (considered as a sister lineage of Pinaceae). In Pinaceae and *Welwitschia*, functional copies of all *ndh* genes were found to be lacking and the loss of *ndh* genes was reported to be initiated with a gene-disrupting inversion in *ndhF* genes [38,39,40,41,42]. Previous studies have also reported the loss of the *ndh* gene family from the chloroplast genome of *Picea* species and the presence of only non-functional *ndh* gene fragments in the plastids [43]. Interestingly, the *ndh* genes which have been reported to be completely lost from the chloroplast genome [44] were not annotated in the present *P. rubens* assembly and only pseudogenes (because of short deletions or insertions) or truncated pseudogenes were retrieved. Moreover, it has also been demonstrated that the plastid *ndh* gene fragments were transferred to the nuclear genome [43], and non-functional plastid *ndh* gene fragments were found to be present in the nuclear genome of *P. abies* [43]. Therefore, annotation of the *ndh* genes in the red spruce chloroplast genome indicates the presence of non-functional pseudogenes or it may be due to the contamination of nuclear DNA [32]. Furthermore, our observation of the lack of a functional copy of *rps16* and the presence of introns in the *clpP* genes in the *P. rubens* chloroplast genome is consistent with such findings in the *Welwitschia* and *Pinus* plastomes [38,39,40]. However, our retrieval of the chlorophyll biosynthesis genes in the chloroplast genome of *P. rubens* is in contrast to the findings in *Welwitschia* where these genes were reported as pseudogenes, missing, or highly divergent [38]. Our assembly and annotation results could be validated in the future with long reads sequencing data of the chloroplast genome of *P. rubens*.

**Figure 2:**
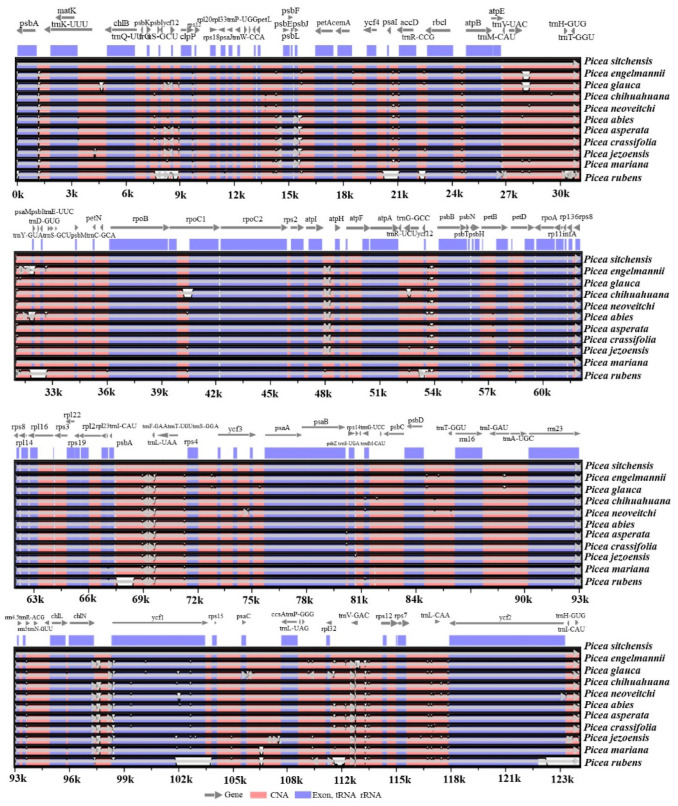
mVISTA-based visual representation of the aligned genomes of the 11 *Picea* species using annotation of the *P. sitchensis* chloroplast genome as the reference.

Among the 11 *Picea* species targeted for global pairwise chloroplast genome sequence alignment and phylogenetic analysis, *P. neoveitchii*, has the largest genome of 124,234 bp followed by *P. jezoensis* (124,146 bp) and *P. asperata* (124,145 bp) and all the five native spruce species of Canada have comparatively shorter genomes (Supplementary Table S1). Global alignment (Shuffle-LAGAN) between chloroplast genomes of these spruce species revealed high synteny. The coding regions were found more conserved in comparison to the non-coding regions. The divergent regions identified in the mVISTA analysis can also be used for the development of useful molecular markers. Furthermore, using the *P. sitchensis* chloroplast genome annotation to plot the sequence identity in the chloroplast genomes of other 10 species including *P. rubens*, a few gaps were observed in the *P. rubens* genome (Figure 2). This was because of the near complete *P. rubens* genome assembly. Overall, these results suggest high synteny and conservation of the chloroplast genomes of the 11 *Picea* species, which is consistent with the generally known evolutionary conservation of the chloroplast genome. These results suggest that the studied *Picea* species are monophyletic and likely originated from a common ancestor. Our results are consistent with the well-known monophyletic origin of *Picea* [17].

The number of genes (107) annotated in the *P. rubens* chloroplast genome was the same as in *P. jezoensis* (107), lower than that annotated in the chloroplast genomes of *P. sitchensis* (114), *P. mariana* (114), *P. glauca* (114), *P. engelmannii* (114), *P. neoveitchii* (116), *P. asperata* (108), *P. crassifolia* (108), and *P. abies* (108), and higher than that annotated in *P. chihuahuana* (89). The protein-coding genes annotated were lower, 69 vs. 73 each in *P. mariana*, *P. glauca,* and *P. engelmanii*, and 72 in *P. abies*, *P. asperata*, *P. crassifolia* [17,18,19,20,21] (Supplementary Table S1). Thus, a somewhat lower number of genes annotated is more likely due to an incomplete genome owing to the very short single-end sequence reads used in our study and less likely due to inherent differences in the chloroplast genome structure between *P. rubens* and 10 other *Picea* species, although the existence of some inherent differences cannot be ruled out. Longer, paired-end sequences (150 bp) were used for the assembly and annotation of the chloroplast genomes of all other *Picea* species [18,19,20,21,22]. It is worth noting that with single-end Illumina sequence reads, a near-complete draft chloroplast genome of *P. rubens* could be assembled and annotated. This draft genome provides a good foundation for improving and finalizing the assembly and annotation of the *P. rubens* chloroplast genome in the future using longer and more modern sequence technologies. Also, the genome resource developed here could potentially be used for various population and evolutionary genetics studies, including the development of cpDNA markers.

### 2.2. SSR Identification and Primer Designing

SSRs or microsatellites are co-dominant and highly polymorphic molecular genetic markers, widely used, especially for population, evolutionary, and conservation genetics studies and forensics. Chloroplast microsatellites have been extensively used for phylogenetic, phylogeography, and biogeography studies. Forty-two SSRs were identified in the *P. rubens* chloroplast genome using MISA [45], of which 27 were mononucleotide, 10 dinucleotide, 1 trinucleotide, 3 tetranucleotide, and 1 hexanucleotide repeat types (Table 3). No pentanucleotide repeats were identified. The mononucleotide A/T repeats followed by the dinucleotide AT repeats were most abundant, which were mostly located in the non-coding regions. One dinucleotide repeat (CT), repeated seven times, was found at the end of the annotated tRNA *trnR-UCU*, and a tetranucleotide (AGGT) repeat, repeated four times, was identified in the annotated ribosomal gene *rrn23*. In these coding regions, length mutation in any of the non-triplet microsatellites like in di and tetranucleotide repeats might result in a frameshift mutation and loss of function. The mutations in these repeats are among the major causes of pseudogene formation [44]. Primer pairs for 26 microsatellite loci were successfully designed for the identified SSRs (Table 4). Microsatellite markers developed from the chloroplast genome sequence of *Pinus thunbergii* [46] have often been used in *Pinus* and *Picea*. However, their cross-species amplification and polymorphism in *Picea* have been low. For example, in *P. rubens* out of 20 markers, only three were found to be polymorphic (Rajora lab). Microsatellites identified in our present study should provide more informative markers for various studies in *P. rubens*, which may be used in other spruce species.

**Figure 3:**
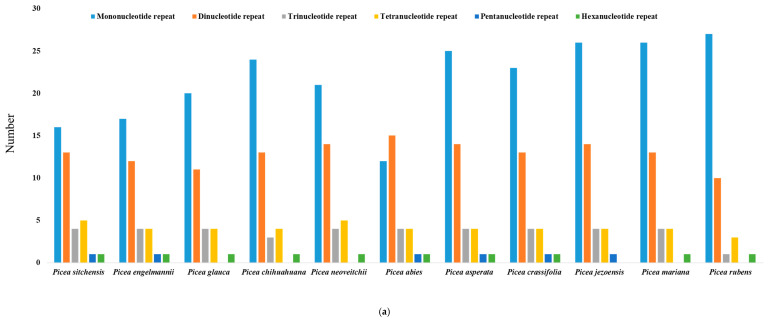

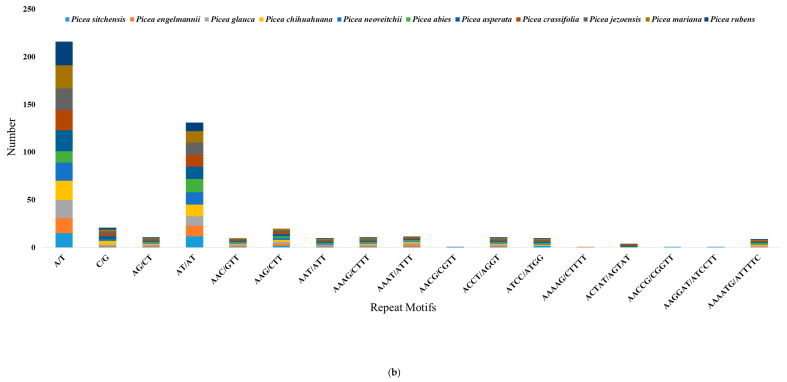
(**a**) Patterns of the number of microsatellites repeats in the chloroplast genomes of *Picea sitchensis*, *P. engelmannii*, *P. glauca*, *P. chihuahuana*, *P. neoveitchii*, *P. abies*, *P. asperata*, *P. crassifolia*, *P. jezoensis*, *P. mariana*, and *P. rubens*. (**b**) Repeat motif distribution in the chloroplast genomes of Picea sitchensis, *P. engelmannii*, *P. glauca*, *P. chihuahuana*, *P. neoveitchii*, *P. abies*, *P. asperata*, *P. crassifolia*, *P. jezoensis*, *P. mariana*, and *P. rubens*.

Our search for microsatellites in the chloroplast genomes using the same criteria as used in *P. rubens* revealed very similar patterns of microsatellite repeats in the chloroplast genomes of other 10 *Picea* species (Table 5; Figure 3a,b; Supplementary Tables S2 and S3). The highest SSR repeats were identified in *P. asperata* (49) and *P. jezoensis* (49), and the lowest in *P. abies* (37) (Table 5). Mononucleotide repeats (A/T) were the most abundant except for the chloroplast genome of *P. abies*, where dinucleotide (AT) repeat was the most abundant (Figure 3a,b). Among the dinucleotide repeats, (AT) repeat was the most abundant in the chloroplast genomes of all 11 *Picea* species (Figure 3b). In *P. abies*, (C/G) mononucleotide repeat was not identified, however, in *P. chihuahuana* four of these repeats were retrieved (Figure 3b). With the SSR search criteria used in our study, only one pentanucleotide repeat was identified in the chloroplast genomes of six *Picea* species (*P. sitchensis*, *P. engelmannii*, *P. abies*, *P. asperata*, *P. crassifolia,* and *P. jezoensis*) and it was absent in other five species (*P. glauca*, *P. chihuahuana*, *P. neoveitchii*, *P. mariana and P. rubens*) (Table 5, Figure 3a,b, Supplementary Tables S2 and S3). Comparative analysis of the SSR repeats in the 11 *Picea* species revealed a similar pattern of microsatellite repeats occurrence in their chloroplast genomes. Our study shows that the mononucleotide (A/T) repeat is most abundant in 10 of the 11 *Picea* species and the dinucleotide (AT) repeat is second most abundant in all 11 *Picea* species chloroplast genomes. Thus, the markers developed from these microsatellite repeats in *P. rubens* may potentially be used for various studies in *P. mariana*, *P. glauca*, *P. sitchensis*, *P. abies*, *P. engelmannii*, *P. chihuahuana*, *P. neoveitchii*, *P. asperata*, *P. crassifolia*, and *P. jezoensis*.

### 2.3. Phylogenetic Analysis

**Figure 4:**
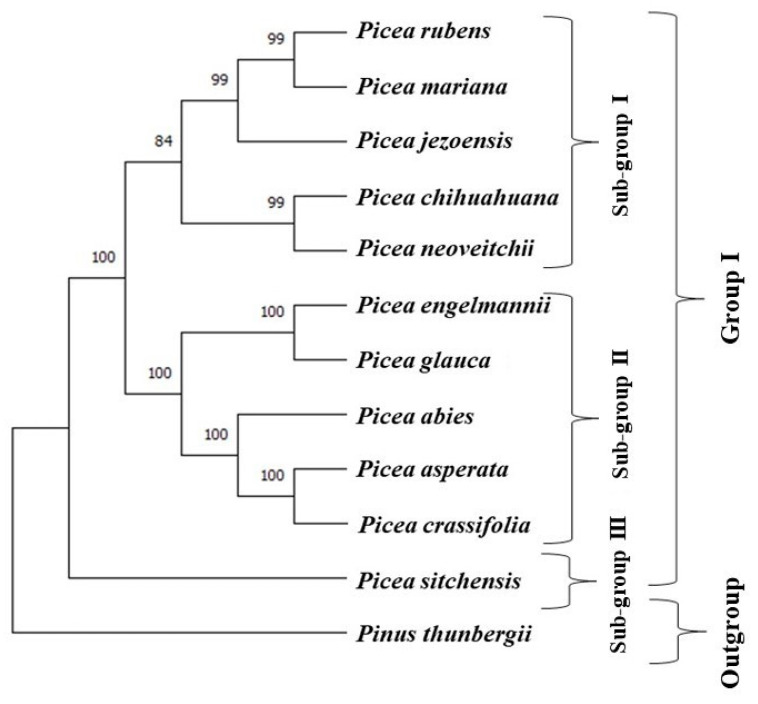
A rooted neighbor-joining phylogenetic tree of 11 *Picea* species using *Pinus thunbergii* as the outgroup, based on their whole chloroplast genome sequences. The numbers on the nodes are the percent support from 1000 bootstraps.

The rooted neighbor-joining tree based on the chloroplast genome sequences revealed one major group of 11 *Picea* species and one outgroup representing *Pinus thunbergii* (Figure 4). The major group I consisted of three sub-groups (Sub-group I, II, and III). Each of the sub-groups I and II had five species each. Subgroup I had a cluster of *P. rubens*, *P. mariana*, *P. jezoensis*, *P. chihuahuana*, and *P. jezoensis*, whereas, in the sub-group II, *P. engelmannii*, *P. glauca*, *P. abies*, *P. asperata*, and *P. crassifolia* clustered together. *P. sitchensis* formed a basal sub-group III (Figure 4). The unrooted tree without the outgroup *P. thunbergii*, except for the position of *P. sitchensis*, displayed the same groupings of the Picea species (Supplementary Figure S2). In the sub-group-I, *P. chihuahuana*, a morphologically distinct and reproductively isolated species was found closely related to *P. neoveitchii*, an endemic and endangered species of China [47]. The sub-group I was further clustered into two sub-groups. *P. mariana*, clustered closely together in the same clade as P. rubens (Figure 4), further supports the fact that both species are closely related [28,30,35,48]. The clustering of *P. jezoensis* in the same clade wherein *P. mariana* and *P. rubens* were present with 99% bootstrap values supports high similarity in the chloroplast genomes of these species. The high chloroplast genome similarities between *P. mariana* and *P. rubens* are consistent with their known high genetic and interspecific crossability relationships [28,30,34,35]. The grouping of *P. jezoensis* with *P. mariana* and *P. rubens* is consistent with their grouping in the same clade based on a few selected chloroplast, mitochondrial and nuclear genes, and/or intergenic spacers and introns [15,17,49]. Furthermore, in sub-group II, *P. glauca* and *P. engelmannii* were found clustered closely together, which suggests a high genetic similarity between these species on the basis of their chloroplast genome sequences. Our results are consistent with high morphological, reproductive, and genetic relationships between these species [50]. These species were also found clustered together in the same clade in previously reported molecular phylogenies of *Picea* [15,17,49,51]. Indeed, these two species hybridize in nature and are mixed up and their species complex is known as interior spruce in British Columbia, Canada. Rajora and Dancik (2000) suggested that these two species could be considered as sub-species of *P. glauca* [50]. The clustering of *P. abies*, *P. crassifolia*, and *P. asperata* in one sub-group suggests their close relationships and is consistent with their clustering in the same group in previous studies based on chloroplast, mitochondrial and nuclear genes, and other DNA elements [15,17,49,51]. The basal position of *P. sitchensis* is consistent with similar results in previous molecular phylogenetic analyses [17,49]. The origin and evolution of *Picea* species are not well understood and there are various hypotheses. The North American origin of the *Picea* hypothesis has been supported by chloroplast DNA-RFLP and *trnC-trnD* and *trnT-trnF-based* phylogenetic studies [17,49]. The basal position of *P. sitchensis* suggests that it may be among the ancestral *Picea* species.

Our study provides the first glimpse of phylogenetic relationships among 11 *Picea* species based on their whole chloroplast genomes. Overall, the phylogenetic relationships in our study are consistent with those previously reported phylogenetic relationships based on biogeographical analysis, chloroplast, mitochondrial, and nuclear genes, and/or intergenic spacers and introns [15,17,49,51]. *Picea* is an important but complex genus with high species diversity and interspecific introgressive hybridization. Our study provides additional insights into the phylogenetic relationships of 11 *Picea* species, which should help in understanding biogeographical patterns and evolution in the genus *Picea*. When the chloroplast genomes of all *Picea* species are available, it will be worthwhile to undertake an evolutionary and phylogenetic analysis based on whole chloroplast genome sequences. We have taken the first step in this direction.

## 3. Materials and Methods

### 3.1. Chloroplast Isolation and DNA Extraction

A *P. rubens* genotype from the West Virginia provenance (S.2020) located in a provenance trial at the Acadian Research Forest near Fredericton, NB, Canada, was used for the isolation of chloroplasts and chloroplast DNA. The branches with needles were collected and kept in dark for 48 h with their cut ends placed in water. A total of 20 g of needles were ground in liquid nitrogen till fine powder and 50 mL of the grinding buffer (Supplementary Table S4) was added to it. After mixing, it was filtered through MIRA cloth into a 50 mL falcon tube followed by spin at 200× *g* for 3 min at 4 °C. The supernatant was transferred to a fresh tube and centrifuged at 1000× *g* for 10 min at 4 °C. Further, the supernatant was discarded, and the pellet was re-suspended in 45 mL wash buffer (Supplementary Table S4) following centrifugation at 1000× *g* for 10 min at 4 °C. The pellet was resuspended in a minimal volume of wash buffer (2 mL) using a Potter-Elvehjem homogenizer with careful pipetting of chloroplasts onto a sucrose gradient prepared using wash buffer (15 mL of 60%, 45%, and 20% sucrose) (Supplementary Table S4) following centrifugation at 7000× *g* for 30 min at 4 °C. The green bands were collected between the 45% and 20% sucrose gradient with the help of a glass Pasteur pipette, and 40 mL of the wash buffer was added to it following centrifugation at 1000× *g* for 10 min at 4 °C. Finally, the supernatant was discarded, and chloroplasts were collected and further used for chloroplast DNA isolation using Cetyltrimethyl Ammonium Bromide (CTAB) method [52]. The quality and quantity of the isolated chloroplast DNA were determined by electrophoresing on ethidium bromide-stained agarose gel.

### 3.2. Library Preparation and Sequencing

The chloroplast genome sequencing was performed in 2009 at the Institute of Applied Genomics, University of Udine. The isolated enriched chloroplast DNA was processed using a DNA sample prep kit coupled with the multiplex sample preparation protocol (Illumina, Inc., San Diego, CA, USA). The DNA was briefly fragmented into small fragments using nebulization following standard blunt-ending and add “A” was performed. The adapters were ligated to the ends of the DNA fragments and a purification step was performed to remove the non-ligated adapters. Further, size selection in the range of 200–250 bp of the adapter-ligated library was performed on a low-range agarose gel following PCR amplification to selectively enrich the DNA fragments with adapters on both ends. The quantity of the prepared library was estimated using Qubit 2.0 Fluorometer (Invitrogen, Carlsbad, CA, USA) and the quality was tested by Agilent 2100 Bioanalyzer High Sensitivity DNA assay (Agilent Technologies, Santa Clara, CA, USA). The library was loaded onto Illumina c-Bot Cluster Station following the manufacturer’s protocol and sequenced with single-end 44 bp reads on Illumina Genome Analyzer II (GAII, Illumina Inc.). Base calling and error estimation was performed using Illumina/Solexa Pipeline (version 1.4). Furthermore, Perl scripts were used to sort and bin all sequences using 4, out of the 12, six nucleotide Illumina indexes. These high-quality single-end reads were used for the final assembly of the chloroplast genome of *P. rubens*.

### 3.3. Chloroplast Genome Assembly, Annotation, and Sequence Architecture

The quality control check of the generated reads was performed using FastQC, and high-quality 44 bp single-end sequencing reads were assembled using SOAPdenovo2 [53,54]. All the assembled contigs were aligned to the reference *P. mariana* chloroplast genome (genotype 40-10-1 and GenBank accession number MT261462) and chloroplast-specific sequences were extracted using BWA-MEM Version 0.7.17.2 and were used for final scaffolding of the *P. rubens* chloroplast genome [55]. The assembled contigs were also mapped to the *P. mariana* reference genome using Geneious Prime [https://www.geneious.com/ (accessed on 17 October 2022) to identify the missing regions in the chloroplast genome of *P. rubens*. We have used the *P. mariana* chloroplast genome as the reference because *P. rubens* and *P. mariana* have high genetic similarities [34,35], although different in their ecological characteristics, for example, *P. mariana* is an early successional species, whereas *P. rubens* is a late successional species. Further, the scaffolding of the assembled contigs was performed using ntJoin v1.0.1 via supplying *P. mariana* as the reference genome with settings as reference_weight = 2 [48]. Finally, the remaining gaps in the scaffolds were filled using Sealer v2.2.3 and multiple values of *k* (*k* = 30 to 90) [56]. The genome assembly quality was estimated using QUAST (Quality Assessment Tools for Genome Assemblies) version 5.0.2 [31]. The assembled chloroplast genome of *P. rubens* was annotated using GeSeq v1.79 [(https://chlorobox.mpimp-golm.mpg.de/geseq.html (accessed on 9 May 2022)] and chloroplast genome sequences of 11 Picea species (*P. mariana*, *P. glauca*, *P. sitchensis*, *P. engelmannii*, *P. abies*, *P. chihuahuana*, *P. morrisonicola*, *P. neoveitchii*, *P. asperata*, *P. crassifolia*, and *P. jezoensis*) as the reference from GenBank [57]. The GeSeq tool helps in the rapid and accurate annotation of chloroplast genomes. This tool combines batch processing with easy selection of the chloroplast reference genome sequences. For annotation, it provides a database of manually organized reference sequences. Moreover, this web-based application uses BLAT-based homology search for genes identification, HMM (Hidden Markov Model) for protein searches, and rRNA identification. Further, for tRNA annotation, the tool uses two *de-novo*-based predictors. Manual correction of the annotation was performed, and the complete *P. rubens* chloroplast genome sequence was submitted to GenBank (accession number OP787482). The circular genome of *P. rubens* chloroplast was obtained using OGDRAW (OrganellarGenomeDRAW) version 1.3.1 [https://chlorobox.mpimp-golm.mpg.de/OGDraw.html (accessed on 9 May 2022)] [58].

The complete chloroplast genomes of 10 *Picea* species and *Pinus thunbergii* (NC_001631.1) were downloaded from NCBI viz., *P. sitchensis* (KU215903.2), *P. engelmannii* (NC_041067.1), *P. glauca* (MK174379.1), *P. chihuahuana* (NC_039584.1), *P. neoveitchii* (NC_043913.1), *P. abies* (NC_021456.1), *P. asperata* (NC_032367.1), *P. crassifolia* (NC_032366.1), *P. jezoensis* (NC_029374.1), *P. mariana* (MT261462.1). Then, global alignment of the entire chloroplast genomes of these 10 *Picea* species and that of *P. rubens* was performed, and comparative genomic divergence was estimated using mVISTA [https://genome.lbl.gov/vista/mvista/submit.shtml (accessed on 17 October 2022)] (Shuffle LAGAN mode) and *P. sitchensis* genome as the reference [59].

### 3.4. Sequence Divergence and Phylogenetic Analysis

The complete chloroplast genomes of the 11 *Picea* species and *Pinus thunbergii* were aligned using MAFFT version 7.471 [60] with default parameters. For the pair-wise sequence divergence, Kimura’s model and to construct the phylogenetic tree, the neighbor-joining (NJ) method with 1000 bootstrap values were implemented in MEGA11 (Mega Evolutionary Genetics Analysis) [61]. *Pinus thunbergii* was used as the outgroup in phylogenetic analysis. Moreover, the InDel polymorphism among these 11 species was estimated using DnaSPv6 [62].

### 3.5. SSR Mining and Primer Designing

The Simple Sequence Repeats (SSRs) were identified in the chloroplast genome of *P. rubens* using MIcroSAtellite (MISA) tool [https://webblast.ipk-gatersleben.de/misa/ (accessed on 9 May 2022)] with search criteria as 10 repeats for mononucleotide, 5 for di, 4 for tri, 3 for tetra, penta and hexanucleotide repeats [45]. A similar SSR search criterion was also used for mining SSR repeats from the chloroplast genomes of 10 other *Picea species* (*P. sitchensis*, *P. engelmannii*, *P. glauca*, *P. chihuahuana*, *P. neoveitchii*, *P. abies*, *P. asperata*, *P. crassifolia*, *P. jezoensis*, and *P. mariana*) for comparative analysis and to understand the SSR repeat pattern in the chloroplast genome sequences of these 10 Picea species and *P. rubens*. Further, Primer3 (https://primer3.ut.ee/ (accessed on 5 October 2022)) [36] was used to design primers from the flanking regions of the identified SSR repeats in the *P. rubens* chloroplast genome.

## 4. Conclusions

We report the first assembly and annotation of the chloroplast genome of *P. rubens* and the first phylogenetic analysis among *Picea* species using the whole chloroplast genome sequences. The short single-end Illumina sequences could be used to assemble near complete draft chloroplast genome in *P. rubens* but longer and/or pair-end sequences are needed to complete and polish the chloroplast genome. The *P. rubens* chloroplast genome has the highest sequence similarities with that of *P. mariana* and the lowest with that of *P. sitchensis.* The mononucleotide (A/T) repeat is most abundant followed by the dinucleotide (AT) repeat in the chloroplast genome of *P. rubens*. The chloroplast genomes of 11 *Picea* species (*Picea sitchensis*, *P. engelmannii*, *P. glauca*, *P. chihuahuana*, *P. neoveitchii*, *P. abies*, *P. asperata*, *P. crassifolia*, *P. jezoensis*, *P. mariana*, and *P. rubens*) have similar patterns of microsatellite repeats occurrence. The global alignment between the chloroplast genomes of these *Picea* species revealed high genome sequence synteny and conservation of coding regions. Our results support a common monophyletic origin of the studied *Picea* species. Our study substantially adds to understanding the phylogeny of *Picea* species. The whole chloroplast genome-based phylogenetic analysis we have reported here may assist in understanding the biogeographical patterns and molecular evolution in *Picea*. Our study provides an important organellar genomic resource for the conifer genomics community. The microsatellites identified in this study may be used for various population and conservation genetics, phylogenetics, phylogeography, and other studies in the genus *Picea* and Pinaceae family.

## Figures and Tables

**Table 1:** QUAST (Quality Assessment Tool for Genome Assemblies) analysis for genome assembly report of the chloroplast genome of *P. rubens* with the chloroplast genomes of *P. sitchensis*, *P. engelmannii*, *P. glauca*, *P. chihuahuana*, *P. neoveitchii*, *P. abies*, *P. asperata*, *P. crassifolia*, *P. jezoensis*, and *P. mariana* as reference.

	*Picea sitchensis*	*Picea engelmannii*	*Picea glauca*	*Picea chihuahuana*	*Picea neoveitchii*	*Picea abies*	*Picea asperata*	*Picea crassifolia*	*Picea jezoensis*	*Picea mariana*
Misassembled contigs length	0	0	0	0	0	0	0	0	0	0
Local misassemblies	1	2	2	2	1	1	1	1	1	0
Mismatches	598	368	368	337	279	270	278	278	131	122
Indels	105	66	72	58	56	72	64	64	36	31
Indels (<=5 bp)	73	52	55	49	44	60	50	50	28	27
Indels (>5 bp)	32	14	17	9	12	12	14	14	8	4
Indels length (bp)	632	493	527	398	238	331	323	321	250	202

**Table 2:** Gene contents of the *P. rubens* chloroplast genome based on genome annotation.

Functional Component	Genes
Photosystem I	*psaA*, *psaB*, *psaC,* and *psaJ*
Photosystem II	*psbA*, *psbB*, *psbC*, *psbD*, *psbE*, *psbF*, *psbH*, *psbJ*, *psbK*, *psbL*, *psbM*, *psbT*, *psbZ,* and *ycf12 (psb30)*
Large ribosomal subunit	*rpl2*, *rpl14*, *rpl16*, *rpl20*, *rpl22*, *rpl23*, *rpl32*, *rpl33*, and *rpl36*
Small ribosomal subunits	*rps2*, *rps3*, *rps4*, *rps7*, *rps8*, *rps11*, *rps12*, *rps14*, *rps15*, *rps18*, and *rps19*
Subunits of cytochrome b/f complex	*petA*, *petB*, *petD*, *petG, petL,* and *petN*
ATP synthase (subunits)	*atpA*, *atpB*, *atpE*, *atpF*, *atpH*, and *atpI*
RNA polymerase	*rpoA*, *rpoB*, *rpoC1*, and *rpoC2*
Chlorophyll biosynthesis genes	*chl*B, *chl*N, and *chl*L
Protease	*clpP*
Maturase	*matK*
Envelope membrane protein	*cemA*
Translation initiation factor	*infA*
Cytochrome c biogenesis	*ccsA*
Subunit Acetyl-CoA-Carboxylate	*accD*
Subunit of Rubisco	*rbcL*
Hypothetical open reading frames	*pafI (ycf3)*, *pafII (ycf4)*, *ycf1*, *ycf2*, and *ycf68*
Ribosomal RNAs	*rrn4.5*, *rrn5*, *rrn16,* and *rrn23*
Transfer RNA	*trnV-UAC/trnY-AUA*, *trnM-CAU*, *trnW-CCA*, *trnP-UGG*, *trnQ-UUG*, *trnK-UUU*, *trnL-CAA*, *trnV-GAC*, *trnP-GGG*, *trnL-UAG*, *trnN-GUU*, *trnR-ACG*, *trnA-UGC*, *trnI-GAU*, *trnT-GGU*, *trnS-UGA*, *trnG-GCC*, *trnF-CAU/trnM-CAU*, *trnS-GGA*, *trnT-UGU*, *trnL/trnL-UAA/UAG*, *trnF-GAA*, *trnG-GCC*, *trnR-UCU*, *trnC-GCA*, *trnD-GUC*, *trnY-GUA*, and *trnE-UUC*

**Table 3:** Type of microsatellite repeat motifs identified in the chloroplast genome of *Picea rubens*.

Repeats	Total Number Identified
A/T	25
C/G	2
AG/CT	1
AT/AT	9
AAT/ATT	1
AAAG/CTTT	1
ACCT/AGGT	1
ATCC/ATGG	1
AAAATG/ATTTTC	1

**Table 4:** Microsatellite loci, primer sequences designed from the flanking region of the SSR sequences identified, and annealing temperatures (Tm).

Locus	Product Size (bp)	Type of Repeat	Length	Tm	Orientation	Primer Sequence (5′-3′)
*RPRSCP1*	167	(A)13	20	55.01	Forward	ATCGGAAGATCCTCTTTTTC
			20	54.95	Reverse	AGCTGTATTGTATGCGGAAT
*RPRSCP2*	176	(TA)8	20	54.15	Forward	TAAGGTGGTAACTCCCATTC
			20	54.73	Reverse	AACAAGAGGATTGGTTCTCA
*RPRSCP3*	241	(TA)5	20	54.90	Forward	GTTAATGAAAGAGCCCAATG
			20	54.62	Reverse	CCATCGATCTTGATAAGGAC
*RPRSCP4*	229	(T)13	20	55.12	Forward	GAAGTATCTGTCCGATCCAA
			20	54.35	Reverse	GTTCCGAACTAGACGATGTT
*RPRSCP5*	250	(TA)5	20	56.03	Forward	ACAGAATCGTGGTGAATCAG
			20	54.91	Reverse	GGATAGCGAGTATTGTCCAG
*RPRSCP6*	194	(AT)7	20	54.94	Forward	GTCTCTCTTCAGAGCGAAAA
			20	55.00	Reverse	GTACCCCGTGATCTCAATAA
*RPRSCP7*	163	(AT)5	20	55.02	Forward	GTAAACCAAGAAGCCCCTAT
			20	55.02	Reverse	CTTCTTCCATTTCTCGATTG
*RPRSCP8*	202	(CT)7	20	54.97	Forward	CAGGAAAAAGAGCTGAAGAA
			20	55.05	Reverse	AGGGTAGATCGGGATAATGT
*RPRSCP9*	231	(A)11	20	55.03	Forward	CCAATCCAATGTGAGAAAGT
			20	54.95	Reverse	CATTGGATCAAGAACAGGAT
*RPRSCP10*	207	(T)15	20	54.44	Forward	TTTCCTTAGTTTCCATCGAC
			20	54.40	Reverse	CGAGAAAGGTGTTTGGTAAT
*RPRSCP11*	236	(T)14	20	55.06	Forward	CATTGCAGGTACAATGACAG
			20	54.89	Reverse	TCGGAAGAGGAATAGGTACA
*RPRSCP12*	245	(T)14	20	55.07	Forward	CAGAGGTCAATTTCTTCTGC
			20	54.82	Reverse	GAAAAAGGAGGAAAGAGAGG
*RPRSCP13*	213	(T)16	20	54.79	Forward	GATGGCTAGAGATTCATTGG
			20	55.23	Reverse	ATTGAGCTGACATCCGTTAC
*RPRSCP14*	234	(T)12	20	54.96	Forward	AACAGGTATGGTTGGTATCG
			20	55.21	Reverse	AGCCGAGCTATTCTCTTTTT
*RPRSCP15*	360	(C)10	20	55.03	Forward	TATCTGATCCTCGAATCACC
			20	55.11	Reverse	ATCGGACCACGATGTAGTAG
*RPRSCP16*	214	(T)13	20	54.57	Forward	GTGATCCAAAAGTGAAAACC
			20	55.43	Reverse	CGAATTACGGACAACCTAAA
*RPRSCP17*	229	(AGGT)3	19	54.89	Forward	TGAAGTAACCCATGCCATA
			20	55.12	Reverse	GGAGACCTGTGTTTTTGGTA
*RPRSCP18*	234	(TAT)4	20	55.03	Forward	ACACCCCACCCTAGAGTTAT
			20	55.33	Reverse	GGGCGACTGAGATATTACAA
*RPRSCP19*	249	(AT)4	22	50.33	Forward	CTCCTAGATAAGCTAACAGAGA
			20	56.33	Reverse	TCGAAACTCCTTGTTGATTG
*RPRSCP20*	360	(ATGAA)3	20	55.85	Forward	ACATCGGTGACAAAGATGAC
			20	55.16	Reverse	GTTCTTCTTTCGGAAGTCCT
*RPRSCP21*	221	(T)13	20	55.78	Forward	CGCAGTATGGGTCTAGCTTA
			20	54.92	Reverse	GCAGATATGGGCAAACTAAC
*RPRSCP22*	229	(AT)10	20	53.61	Forward	TCCTTTTCCGTATACTTTCC
			20	54.93	Reverse	CGGGTTAATGTGAGCTTATC
*RPRSCP23*	239	(AAAG)3	20	55.12	Forward	AGGTTCGAGTCAAATAGCAA
			20	55.37	Reverse	AACCGTACATACGACTTTCG
*RPRSCP24*	229	(T1)12	20	55.17	Forward	GGACATGTGGAAAAGAGAAA
			20	55.37	Reverse	GCGCATGTATAAGACCAAAT
*RPRSCP25*	177	(AT)8	21	55.12	Forward	CGATATCAATACTCGAAGACG
			20	54.75	Reverse	TGTCTACCATTTCACCATCA
*RPRSCP26*	210	(T)12	20	55.87	Forward	GATCTCGGAGTGAAGAACCT
			20	54.81	Reverse	GAAAGAGCAATGGAATATGG

**Table 5:** Comparison of the SSRs identified in the chloroplast genome of *P. rubens* with other 10 *Picea* species.

Statistics	*Picea sitchensis*	*Picea engelmannii*	*Picea glauca*	*Picea chihuahuana*	*Picea neoveitchii*	*Picea abies*	*Picea asperata*	*Picea crassifolia*	*Picea jezoensis*	*Picea mariana*	*Picea rubens*
Total size of examined sequences (bp)	124,049	123,542	123,421	123,488	124,234	124,084	124,145	124,126	124,146	123,961	122,115
Total number of identified SSRs	40	39	40	45	45	37	49	46	49	48	42
Number of SSRs present in compound formation	6	4	5	7	7	7	9	7	10	9	8
Number of sequences containing more than 1 SSR	1	1	1	1	1	1	1	1	1	1	1

## Data Availability

The Illumina chloroplast genome sequence data has been submitted to NCBI SRA with accession number PRJNA895863 and the chloroplast genome assembly and annotation data to GenBank with accession number OP787482.

## Supplementary Materials

The following supporting information can be downloaded at: https://www.mdpi.com/article/10.3390/ijms232315243/s1.
