# Supplementary material for: Assembly and Annotation of Red Spruce (Picea rubens) Chloroplast Genome, Identification of Simple Sequence Repeats, and Phylogenetic Analysis in Picea
Source: Int J Mol Sci. 2022 Dec 3;23(23):15243. doi: 10.3390/ijms232315243 (PMC9739956; doi:10.3390/ijms232315243)
Supplement: Supplementary file 1 [file ijms-23-15243-s001.zip › Supplementary Material File S1.pdf]

Supplementary Material File S1

Assembly and Annotation of Red Spruce (*Picea rubens*) Chloroplast Genome, Identification of Simple Sequence Repeats, and Phylogenetic Analysis in *Picea*

Rajni Parmar [1], Federica Cattonaro [2], Carrie Phillips [3], Serguei Vassiliev [4], Michele Morgante [2,5] and Om P. Rajora [1,3,*]

[1] Faculty of Forestry and Environmental Management, University of New Brunswick, 28 Dineen Drive, Fredericton, NB E3B 5A3, Canada
[2] IGA Technology Services, Via Jacopo Linussio, 51, 33100 Udine, Italy
[3] Forest Genetics and Biotechnology Group, Department of Biology, Dalhousie University, Halifax, NS B3H 4J1, Canada
[4] ACENET, University of New Brunswick, Fredericton, NB E3B 5A3, Canada
[5] Laboratory of Plant Genomics, University of Udine, 33100 Udine, Italy
* Correspondence: om.rajora@unb.ca

Supplementary Table S1: Comparison of chloroplast genome assembly and annotation of *Picea rubens* with that of 10 other *Picea* species.

Feature	*Picea sitchensis*	*Picea engelmannii*	*Picea glauca*	*Picea chihuahuana*	*Picea neoveitchii*	*Picea abies*	*Picea asperata*	*Picea crassifolia*	*Picea jezoensis*	*Picea mariana*	*Picea rubens*
**Accession Number**	KU215903.2	NC_041067.1	MK1743 79.1	NC_039584.1	NC_043913.1	NC_02145 6.1	NC_03236 7.1	NC_032366. 1	NC_02937 4.1	MT2614 62.1	OP787482
**Size (bp)**	124,049	123,542	123,421	123,488	124,234	124,084	124,145	124,126	124,146	123,961	122,115
**% GC content**	38.7	38.74	38.74	38.7	38.77	38.72	38.7	38.7	38.8	38.7	38.96
**Number of protein coding genes**	74	74	74	57	77	72	72	72	68	74	69
**Number of rRNAs**	4	4	4	4	4	4	4	4	4	4	4
**Number of tRNAs**	36	36	36	28	35	32	32	32	35	36	28

Supplementary Table S2. The number of mononucleotide to hexanucleotide SSR repeats identified in the chloroplast genomes of *P. rubens* and 10 other *Picea* species.

**Repeat type**	***Picea sitchensis***	***Picea engelmannii***	***Picea glauca***	***Picea chihuahuana***	***Picea neoveitchii***	***Picea abies***	***Picea asperata***	***Picea crassifolia***	***Picea jezoensis***	***Picea mariana***	***Picea rubens***
Mononucleotide	16	17	20	24	21	12	25	23	26	26	27
Dinucleotide	13	12	11	13	14	15	14	13	14	13	10
Trinucleotide	4	4	4	3	4	4	4	4	4	4	1
Tetranucleotide	5	4	4	4	5	4	4	4	4	4	3
Pentanucleotide	1	1	0	0	0	1	1	1	1	0	0
Hexanucleotide	1	1	1	1	1	1	1	1	0	1	1

Supplementary Table S3. SSR repeats and their numbers in the chloroplast genomes of *P. rubens* and 10 other *Picea* species.

**Nucleotide repeat**	***Picea sitchensis***	***Picea engelmannii***	***Picea glauca***	***Picea chihuahuana***	***Picea neoveitchii***	***Picea abies***	***Picea asperata***	***Picea crassifolia***	***Picea jezoensis***	***Picea mariana***	***Picea rubens***
A/T	15	16	19	20	19	12	22	21	23	24	25
C/G	1	1	1	4	2	0	3	2	3	2	2
AG/CT	1	1	1	1	1	1	1	1	1	1	1
AT/AT	12	11	10	12	13	14	13	12	13	12	9
AAC/GTT	1	1	1	1	1	1	1	1	1	1	0
AAG/CTT	2	2	2	2	2	2	2	2	2	2	0
AAT/ATT	1	1	1	0	1	1	1	1	1	1	1
AAAG/CTTT	1	1	1	1	1	1	1	1	1	1	1
AAAT/ATTT	1	2	2	1	1	1	1	1	1	1	0
AACG/CGTT	0	0	0	0	1	0	0	0	0	0	0
ACCT/AGGT	1	1	1	1	1	1	1	1	1	1	1
ATCC/ATGG	2	0	0	1	1	1	1	1	1	1	1
AAAAG/CTTTT	0	1	0	0	0	0	0	0	0	0	0
ACTAT/AGTAT	0	0	0	0	0	1	1	1	1	0	0
AACCG/CGGTT	1	0	0	0	0	0	0	0	0	0	0
AAGGAT/ATCCTT	1	0	0	0	0	0	0	0	0	0	0
AAAATG/ATTTTC	0	1	1	1	1	1	1	1	0	1	1

Supplementary Table S4: Details of grinding and wash buffers and sucrose gradient constituents used for isolation of chloroplasts from needles of *Picea rubens*.

**Grinding buffer constituents**	**Wash buffer constituents**	**Sucrose Gradient:** Prepared using wash buffer for each sample
30mM MOPS (Stock: 0.5M, pH 7.4)	10mM MOPS (Stock: 0.5M, pH 7.4)	15ml 60% sucrose
0.35M mannitol	0.35M mannitol	15ml 45% sucrose
2.5mM EDTA (1M)	1mM EDTA	15ml 20% sucrose
0.5M EDTA	2mM sodium bisulfite	
0.30% PVP25	0.10% BSA	
2mM sodium bisulfite		
0.2 %BSA		
2mM cysteine		

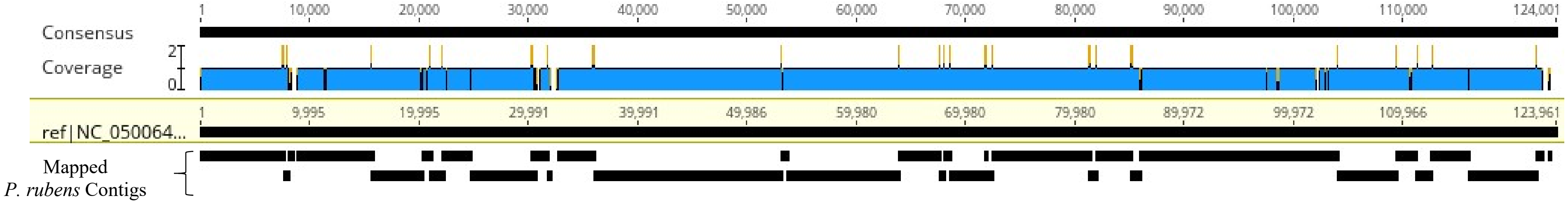

Supplementary Figure S1: Assembled contigs of *P. rubens* mapped to the *P. mariana* chloroplast genomes (reference number: NC_050064.1). The gaps in the bottom two lines represent the missing parts in the *P. rubens* assembly.

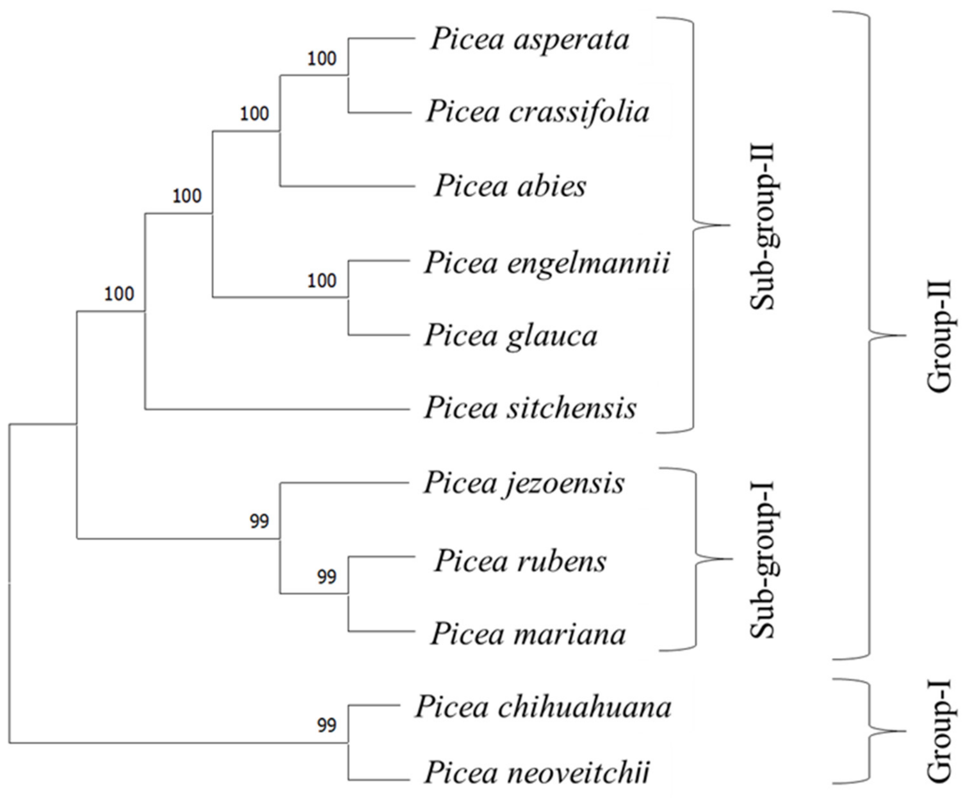

Supplementary Figure S2: An unrooted neighbor-joining phylogenetic tree of 11 *Picea* species based on their total chloroplast genome sequences. The numbers on the nodes are the per-cent support from 1000 bootstraps.
